# Dr. Lippe’s New Evidence for Curantur

**Published:** 1885-02

**Authors:** R. E. Dudgeon

**Affiliations:** London


### DR. LIPPE'S NEW EVIDENCE FOR CURANTUR.

R. E. Dudgeon, M. D., London.

All readers of The Homœopathic Physician who have the slightest sense of humor must feel grateful to Dr. Lippe for the exquisite treat he affords them in his contribution to the Curantur *versus* Curentur controversy in the December number.

Dr. Lippe seriously assumes the judicial function, and, adopting the magniloquent "we," proceeds to "sum up the case" without apparently the slightest misgiving as to the propriety of an avowed partisan setting himself up as the judge in a cause in which he has all along so eagerly taken up one side. But not only does he commit this solecism, he performs the still more unprecedented feat of importing into his quasi-judicial summing up some altogether fresh evidence, never so much as hinted at in any previous phase of the discussion, in support of the side he favors. He says: "It had been found that the curentur *once* found in Hahnemann's writings was owing to the oversight of the proof-reader." On reading this, I felt very much disposed to employ the favorite expression of the charming American young ladies who deign to enliven the gloom of our befogged island and to exclaim, "Oh! my." Is not Dr. Lippe's statement just a trifle too strong, or, perhaps I should say, just a little bit too weak, to go down with the "intelligent reader"? For Dr. Lippe knows, or, being a first-chop Hahnemannian, ought to know, that it is not *once* only that Hahnemann uses the formula, "similia similibus curentur," but no less than *six* times—to wit: in the introduction of all the five editions of the *Organon*, several of which are rewritten from beginning to end, and all of which are very much altered; but the fatal "curentur" stands out conspicuously in all five, as I have ascertained by examining all the editions, three of which, viz.: the first, second, and fifth, are in my own library, where they can be seen by any one who will honor my modest abode with a visit. The sixth instance of "curentur" is in the letter Hahnemann wrote to the French Minister of Public Instruction, which may be read in the *British Journal of Homœopathy*, Vol. XXXVIII, pp. 64–66.

So that *one* careless proof-reader is not enough. Dr. Lippe will have to find *six* careless proof-readers, whose operations range from 1810, the year of the publication of the first edition of the *Organon*, to 1835, the date of the letter above named. Really, his *one* oversighting proof-reader does but little credit to

Dr. Lippe's inventive faculty. While he was inventing proof-readers he might as well have supplied them in sufficient numbers. Why could he not just as well have summoned "from the vasty deep" half a dozen proof-readers to his aid, given their names and addresses, and mentioned the sums paid to them by those arch-traitors, "Drs. Hughes, Dudgeon & Co.," for the purpose of suborning them to corrupt Hahnemann's text? We know how Falstaff multiplied his "men in buckram" to meet the exigencies of his story. What was to hinder Dr. Lippe from following this classical example? When once you begin drawing on your imagination for your facts, it is surely worth while giving your imaginary facts in sufficient numbers to meet the requirements of the case. But probably Hahnemann, like Dr. Lippe and myself and other distinguished authors, was his own proof-reader, and if so, then no doubt he wrote "similia similibus curentur" just in order to spite his devoted disciple, Dr. Lippe.

Would Dr. Lippe desire any further evidence in favor of my contention that Hahnemann wrote "curentur" and not "curantur"? Well, here is a little bit at his service. Mr. Everest, a learned clergyman and enthusiastic advocate of Homœopathy, the author of several excellent popular works on Homœopathy, who prided himself on his pure Hahnemannism and enjoyed the friendship and intimacy of the illustrious founder of Homœopathy during the last few years of his life, in the prefatory remarks to a sermon preached by him in 1851 in aid of the Hahnemann Hospital of London, and published by him in the same year, says: "The great master has placed on that rock this beacon: *Similia similibus curentur*—let like be treated by like. His disciples have translated 'curo' by our word 'to cure,' and changed his advice into the law—'like cures like.'" And again: "Hahnemann knew all this, and instead of saying '*similia similibus sanantur*,' he said '*similia similibus curentur*'—let like be treated by like."

Of course, we all know that behind the therapeutic rule, "let likes be treated by likes," there lies the natural law, "likes are cured by likes," for if we did not believe this, we should not think of treating likes by likes. What I assert is, that Hahnemann's formula is "similia similibus curentur," which is not the statement of a natural law, but a rule for the treatment of disease, as he himself expressly says when he interprets this formula in the first editions of the *Organon* in this way: "In order to effect a mild, rapid, certain, and permanent cure, choose, in every case of disease, a medicine which can of itself produce an affection similar to that it is meant to cure—similia similibus curentur!" That this is a therapeutic rule, and not the expression of a natural law, no one—unless it be Dr. Lippe—can doubt, and that it is *mandatory* in the sense of "containing a command" (as Webster defines the word) surely no one—unless it be Dr. Lippe—would deny. The natural law: like cures like—"similia similibus curantur," like all other natural laws, is, of course, affirmative, and not mandatory at all.

Dr. Lippe, with a naïveté one would hardly look for in such a well-seasoned and experienced controversialist, tries to divert the controversy between us into a different channel by abusing the *British Journal of Homœopathy*, expecting, I suppose, that I should rush to the defense of that defunct periodical. But I am not to be caught by this old trick, which resembles that known in the hunting field as trailing a red herring across the scent. I do not attach sufficient importance to Dr. Lippe's opinions on that or any other subject to care for engaging with him in any controversy of that sort. I only entered the lists against him in defense of the text of Hahnemann's writings. Having done this, I leave him in undisturbed enjoyment of his favorite amusement of denouncing all who differ from him in the peculiar form of courtesy for which he is distinguished, and of which I trust he may long have a monopoly.

As Dr. Lippe is fond of admonishing his colleagues about their "fatal errors," I would recommend to his consideration a few that are conspicuous in his contributions to this discussion: 1st. It is a "fatal error" to hazard a statement respecting an author's words, without referring to the original source. 2d. It is a "fatal error" to employ words in a sense different to that generally received, as in his use of "mandatory." 3d. It is a "fatal error" to affect to assume judicial functions while a declared partisan of one side. 4th. It is a "fatal error" to draw on the imagination for facts, as Dr. Lippe has done in regard to his careless proof-reader. 5th. It is a "fatal error" to attempt to shunt the controversy on to another line by launching out into abuse—say of the *British Journal of Homœopathy*—when the subject under discussion is the correct text of Hahnemann's writings.

These are all "fatal errors" in a disputant, and in future Dr. Lippe would do well to avoid them if he is desirous of acquiring a reputation in this field. In the meantime, I wish him a Merry Christmas, a Happy New Year, a good appetite, and sound sleep, undisturbed by haunting visions of phantom proof-readers.

LONDON, December 16th, 1884.

